# Acetic acid: a cost-effective agent for mitigation of seawater-induced salt toxicity in mung bean

**DOI:** 10.1038/s41598-019-51178-w

**Published:** 2019-10-23

**Authors:** Md. Mezanur Rahman, Mohammad Golam Mostofa, Md. Abiar Rahman, Md. Robyul Islam, Sanjida Sultana Keya, Ashim Kumar Das, Md. Giashuddin Miah, A. Q. M. Robiul Kawser, S. M. Ahsan, Abeer Hashem, Baby Tabassum, Elsayed Fathi Abd_Allah, Lam-Son Phan Tran

**Affiliations:** 1 Department of Agroforestry and Environment, Bangabandhu Sheikh Mujibur Rahman Agricultural University, Gazipur, 1706 Bangladesh; 2 Department of Biochemistry and Molecular Biology, Bangabandhu Sheikh Mujibur Rahman Agricultural University, Gazipur, 1706 Bangladesh; 3 Department of Biotechnology, Bangabandhu Sheikh Mujibur Rahman Agricultural University, Gazipur, 1706 Bangladesh; 4 Department of Aquaculture, Bangabandhu Sheikh Mujibur Rahman Agricultural University, Gazipur, 1706 Bangladesh; 5 Department of Agriculture, Bangabandhu Sheikh Mujibur Rahman Science and Technology University, Gopalganj, Bangladesh; 6 Botany and Microbiology Department, College of Science, King Saud University, P.O. Box. 2460, Riyadh, 11451 Saudi Arabia; 7 Mycology and Plant Disease Survey Department, Plant Pathology Research Institute, ARC, Giza, 12511 Egypt; 8 Toxicology Laboratory, Department of Zoology, Govt. Raza P.G. College, Rampur, UP 244091 India; 9 Plant Production Department, College of Food and Agricultural Sciences, King Saud University, P.O. Box. 2460, Riyadh, 11451 Saudi Arabia; 10 Institute of Research and Development, Duy Tan University, 03 Quang Trung, Da Nang, Vietnam; 11 Stress Adaptation Research Unit, RIKEN Center for Sustainable Resource Science, Yokohama, Japan

**Keywords:** Plant physiology, Salt

## Abstract

The current study sought the effective mitigation measure of seawater-induced damage to mung bean plants by exploring the potential roles of acetic acid (AA). Principal component analysis (PCA) revealed that foliar application of AA under control conditions improved mung bean growth, which was interlinked to enhanced levels of photosynthetic rate and pigments, improved water status and increased uptake of K^+^, in comparison with water-sprayed control. Mung bean plants exposed to salinity exhibited reduced growth and biomass production, which was emphatically correlated with increased accumulations of Na^+^, reactive oxygen species and malondialdehyde, and impaired photosynthesis, as evidenced by PCA and heatmap clustering. AA supplementation ameliorated the toxic effects of seawater, and improved the growth performance of salinity-exposed mung bean. AA potentiated several physio-biochemical mechanisms that were connected to increased uptake of Ca^2+^ and Mg^2+^, reduced accumulation of toxic Na^+^, improved water use efficiency, enhanced accumulations of proline, total free amino acids and soluble sugars, increased catalase activity, and heightened levels of phenolics and flavonoids. Collectively, our results provided new insights into AA-mediated protective mechanisms against salinity in mung bean, thereby proposing AA as a potential and cost-effective chemical for the management of salt-induced toxicity in mung bean, and perhaps in other cash crops.

## Introduction

Environmental pollution, including soil pollution, is a great threat to world agriculture, endangering sustainable productivity and future food supply^1^. Soil pollution, resulted from extensive use of salt-rich water for irrigation and seasonal intrusion of saline water to the coastal lowlands, has now been recognized as a massive cause of losing soil quality and agricultural output in many areas of the world, especially those adjacent to the seas^2–4^. Furthermore, climate-driven rises in temperature and scarcity of rainfall leading to drier climate and extra vaporization of soil water exacerbate salinity curse on agriculture^4^, causing yield reduction in many crops that are vital for world’s food security. Therefore, development of effective strategies for efficient mitigation of soil salinity effects on crop productivity is crucial for sustainable agriculture and food security.

Under salt stress conditions, plants accumulate excessive sodium (Na^+^) and chloride (Cl^−^) ions that are responsible for different physiological and biochemical abnormalities, including ionic imbalance, impaired gas exchange parameters, disturbed water homeostasis and accumulation of reactive oxygen species (ROS)^5–7^. To counteract the detrimental effects of salt-induced stress, plants adopt some key mechanisms, including (i) exclusion and compartmentation of Na^+^ and Cl^−^ ions into vacuoles or old tissues to reduce their toxic effects, (ii) production of compatible solutes like proline (Pro), free amino acids and sugars to minimize high salinity-caused osmotic stress, (iii) activation of both non-enzymatic [e.g. phenolic compounds, carotenoids, flavonoids, ascorbate (AsA) and glutathione (GSH)] and enzymatic (e.g. superoxide dismutase, SOD; catalase, CAT; ascorbate peroxidase, APX; glutathione *S*-transferase, GST and glutathione peroxidase, GPX) antioxidant systems to detoxify ROS and prevent cells from oxidative damage^5,7–9^.

In Bangladesh, soil salinity is progressively increasing in terms of magnitude and affected land, resulting in 26.73% augmentation of salt-prone areas from 1973 to 2009^10^. Salinity situation in Bangladesh has been predicted to become much worse, and the annual median, wet-season and dry-season soil salinity may rise to 39.2, 36.6 and 13.1%, respectively, by the year 2050, compared with the base year 2009^11^. Between 2000 and 2009, salt-water intrusion crept 15 kilometers north of the southern coast, and has recently reached to 160 kilometers, threatening 40% farmland in southern Bangladesh^12^. Additionally, most of the agricultural land in the southern coastal part of Bangladesh remains untenable for agriculture during the drying season because of practicing saline water irrigation^13,14^. Eventually, nearly 12 millions out of 37 million people live below the poverty line in coastal districts of Bangladesh^15^, leading to the aggravation of maternal and childhood undernutrition^16^. Due to socio-economic constraints, a large number of people in the coastal areas have to rely on vegetable proteins, especially on pulse proteins due to their low price relative to animal proteins^17^.

In Bangladesh, mung bean (*Vigna radiata*) is an important pulse crop, representing a major source of pulse proteins for the countrymen. It has been estimated that mung bean cultivation covered approximately 0.3188 million hectares (Mha) out of 0.9976 Mha of total pulse crop areas in the years 2016–17, and produced 0.21 million metric tonnes of mung bean^18^. Inclusion of mung bean as a cash crop into the major rice-based cropping system in the coastal regions of Bangladesh is increasing day-by-day^19^, because its cultivation requires less fertilizer and pesticides, and thus ensures farmers good economic benefits and nutritional security^20^. Unfortunately, coastal salinity has arisen as a great curse on mung bean cultivation in coastal lands, thereby threatening sustainable agricultural output in southern part of Bangladesh. At present, nearly 24% of coastal areas are not suitable for mung bean production, whereas only 14 and 19% of coastal lands are marginally and moderately suitable, respectively (http://cropzoning.barcapps.gov.bd/homes/bdMap/6). Therefore, the country needs to find a solution for efficient alleviation of the effects of soil salinity to enhance mung bean productivity in salt-affected coastal areas.

Although biotechnological approach is promising in dealing with salt stress^8,21,22^, simple and less expensive technologies should also be developed for low-income countries like Bangladesh where inadequate investment in research and development does not enable scientists to research and develop genetically modified crops. In this context, exploration of the potential roles of exogenous chemicals, including signaling molecules, may provide an effective solution for the improvement of plant resiliency toward the adverse effects of ever-changing environmental assaults^23–25^. Acetic acid (AA) has recently come into limelight because of the finding on its involvement in enhancement of drought tolerance in *Arabidopsis thaliana*, rapeseed (*Brassica napus*), maize (*Zea mays*), wheat (*Triticum aestivum*) and rice (*Oryza sativa*)^26^. With this clue, we also anticipate that this cost-effective and easily accessible AA might be a cutting-edge solution in the management of salt stress in a prospective crop mung bean in order to enhance its cultivation and production in coastal areas of Bangladesh. Moreover, the intrinsic mechanisms AA uses to confer plant tolerance to salinity, if it does at all, are still elusive, thus requiring in-depth investigations using a variety of crop species.

In the current study, we first examined whether AA provides protection to mung bean under seawater-induced salinity, and to our expectation it really did. Next, we also examined the regulatory roles of AA in improving mung bean tolerance to salinity by investigating the morpho-physiological and biochemical mechanisms through assessments of the following key attributes: (i) plant growth performance and biomass production, (ii) Na^+^ accumulation, (iii) ionic balance, (iv) photosynthetic features, (v) photosynthetic pigment status, (vi) excessive salinity-induced oxidative damage in terms of elevated ROS levels and lipid peroxidation, (vii) improvement of antioxidant defense, and (viii) different types of osmolyte accumulations.

## Methods

### Plant growth conditions and salt treatments

BU Mung-5, a short-duration (50–55 days to maturity) and high-yielding (1.5–1.8 tonnes ha^−1^) mung bean (*Vigna radiata*) variety, was used in the present study. Healthy seeds of BU Mung-5 were subjected to surface-sterilization using a solution of 5% (v/v) sodium hypochlorite and 0.2% (v/v) Tween-20 for 20 min followed by 3-times washing with distilled water (dH_2_O). Subsequently, the sterilized seeds were soaked in dH_2_O and kept in dark for 8 h at room temperature to fasten the germination process. Afterwards, twenty seeds were planted in each plastic pot (height × diameter = 17 cm × 18 cm) containing 2.5 Kg soil per pot. The soil used was prepared by mixing cow dung, sand and soil at the ratio of 1:0.5:2 (in weight basis). Furadan (a well-known pesticide) was mixed with the soil (3.0 g Kg^−1^ of soil) during soil preparation to curtail the growth of soil-borne pathogens. Urea (diluted in water at 4.0 g L^−1^) was applied to the pots at day 10^th^ after sowing to assure the appropriate supply of nitrogen, and the number of seedlings was thinned to 12 in each pot.

Sixteen-day-old seedlings at vegetative V1 stage (fully developed first trifoliolate) were grouped into six sets, where two sets in each case were irrigated with tap water, 8 dS m^−1^ and 16 dS m^−1^ of saline water (200 mL to each pot) at one-day-interval for a duration of 14 days (7 times in total). In parallel, one set of 3 pots (one pot from each irrigation group) was subjected to foliar application (30 mL to each pot) with acetic acid (AA, 20 mM) during 11:30 AM to 12:00 PM, and the remaining set of 3 pots was sprayed with tap water only (30 mL to each pot). The surfactant Tween-20 (0.2%, v/v) was added to ensure maximum adherence of AA and water to the leaves. The dose of exogenous AA (20 mM) was selected based on the previous report of Kim *et al*.^26^. Thirty-day-old plants were then harvested to assess various morphological, physiological and biochemical features. The experiment was repeated three times, and the first trifoliate leaves from the bottom of the plants were collected for determination of various physiological and biochemical parameters.

### Determination of growth parameters, electrolyte leakages and relative water contents

Three plants were randomly harvested from each treatment to measure shoot height, and root and shoot fresh weights (FWs) and dry weights (DWs) according to Rahman *et al*.^13^. For measurement of primary root length, control and treated plants were carefully uprooted from the soils, and the roots were immediately washed using 10 mM calcium chloride solution to remove Na^+^ that adhered to the root surface. Afterwards, the length of each primary root (*n*** = **3) was manually measured using a measuring scale. Relative water contents (RWCs) and electrolyte leakages (ELs) of the detached aerial parts of the plants were determined and calculated following the methods of Nishiyama *et al*.^27^.

### Measurement of leaf area and leaf succulence

The first trifoliate leaves (one trifoliolate from each plant) from the bottom were taken from three plants of each treatment for determination of total leaf area per trifoliolate according to Carleton and Foote^28^. Leaf succulence was assessed using 9 leaves obtained from three trifoliolates (three leaves from each first trifoliolate from each plant) from three plants of each treatment. Leaf FW and leaf area of each sample were used to determine leaf succulence according to the equation reported in Silveira *et al*.^29^. Leaf succulence (mg FW cm^−2^) = FW/leaf area.

### Estimation of Na^+^ and mineral nutrient contents in plant tissues

The oven-dried samples of roots and shoots were used for estimating the amounts of Na^+^, potassium (K^+^), calcium (Ca^2+^) and magnesium (Mg^2+^) ions using an atomic absorption spectrophotometer (HITACHI, Model: 170-30, Japan) following the methods of Mostofa *et al*.^30^.

### Gas exchange parameters

Different photosynthetic features, including photosynthetic rate (Pn), stomatal conductance to H_2_O (g_s_), transpiration rate (E) and leaf temperature (LT) were determined with the help of the LI-6400XT system (LI-COR Biosciences, Lincoln, Nebraska, USA) from 11:00 AM to 12:30 PM under full sunlight. Instantaneous water use efficiency (WUEins) and intrinsic water use efficiency (WUEint) were calculated by dividing the Pn with E and g_s_, respectively.

### Photosynthetic pigment, total phenolic and flavonoid contents

The contents of chlorophylls (Chl *a*, *b* and total Chls) and carotenoids (Cars) in fresh mung bean leaves were spectrophotometrically determined according to the following equation proposed by Arnon^31^, and Lichtenthaler and Wellburn^32^, respectively.

$$\begin{array}{rcl}{\rm{Chl}}\,a\,({\rm{mg}}\,{{\rm{g}}}^{-1}\,{\rm{FW}}) & = & (0.0127\times {\rm{D}}663-0.00269\times {\rm{D}}645)\times {\rm{V}}/{\rm{W}}\\ {\rm{Chl}}\,b\,({\rm{mg}}\,{{\rm{g}}}^{-1}\,{\rm{FW}}) & = & (0.0229\times {\rm{D}}645-0.00468\times {\rm{D}}663)\times {\rm{V}}/{\rm{W}}\\ {\rm{Total}}\,{\rm{Chls}}\,({\rm{mg}}\,{{\rm{g}}}^{-1}\,{\rm{FW}}) & = & (20.2\times {\rm{D}}645+8.02\times {\rm{D}}663)\times {\rm{V}}/(1000\times {\rm{W}})\\ {\rm{Carotenoids}}\,({\rm{mg}}\,{{\rm{g}}}^{-1}\,{\rm{FW}}) & = & (1000\times {\rm{A}}470-2.270\times {\rm{Chl}}\,a-81.4\times {\rm{Chl}}\,b/227)\times {\rm{V}}/(1000\times {\rm{W}}),\end{array}$$

where V is the volume of 80% (v/v) acetone (mL), and W is the FW of leaf samples (g).

Total phenolic and total flavonoid contents in leaf tissues were estimated according to the methods of Ainsworth and Gillespie^33^, and Zhishen *et al*.^34^, respectively.

### Superoxide, malondialdehyde and hydrogen peroxide contents

The level of superoxide (O_2_^•−^) in mung bean leaves was determined according to the method of Elstner and Heupel^35^ with minor modifications. Fresh leaf samples (0.3 g) were crushed in 3 mL of potassium phosphate (K-P) buffer (65 mM, pH 7.8) followed by a centrifugation at 5,000 × *g* for 10 min at 4 °C. After collection, 750 µL of the supernatant was added to a reaction mixture containing 675 µL of K-P buffer (65 mM, pH 7.8) and 70 µL of hydroxylamine chlorhydrate (10 mM). After an incubation for 20 min at 25 °C, the solution was mixed with 375 µL of sulfanilamide (17 mM) and 37.5 µL of *α*-naphthylamine (7 mM). The resultant solution was further incubated for 20 min at 25 °C before the addition of 2.25 mL of diethyl ether (v/v). The absorbance was read at 530 nm, and the content of O_2_^•−^ was calculated using a standard graph developed with sodium nitrite. Hydrogen peroxide (H_2_O_2_) and malondialdehyde (MDA) levels in leaf tissues were quantified following the methods of Yu *et al*.^36^, and Heath and Packer^37^, respectively.

### Histochemical analyses of ROS production and root membrane integrity

Visual detection of O_2_^•−^ and H_2_O_2_ in mung bean leaves was carried out according to the methods of Mostofa and Fujita^38^. Membrane integrity of mung bean roots was histochemically assessed using Schiff’s reagent following the method of Srivastava *et al*.^39^.

### Enzyme extraction and the assessment of enzyme activities

For analyzing the activities of various enzymes, leaf samples (0.5 g/sample) were crushed in 1 mL of 50 mM ice-cold K-P buffer (pH 7.0), which contained potassium chloride (100 mM), ascorbate (AsA, 1 mM), *β*-mercaptoethanol (5 mM) and glycerol (10%; v/v). The homogenized plant material was centrifugated at 11,500 × *g* for 12 min^38^, and the supernatant was collected for estimating enzyme activities.

The activity of SOD (EC 1.15.1.1) was determined based on the reaction of xanthine/xanthine oxidase system^40^. The reaction mixture contained enzyme solution (5 µL), K-P buffer (50 mM, pH 7.0), nitro-blue tetrazolium (NBT, 2.24 mM), 0.1 unit catalase, xanthine (2.36 mM) and 0.1 unit of xanthine oxidase (final volume 700 µL). The spectrophotometric reading was recorded for the change of absorbance observed for 1 min at 560 nm, and SOD activity was calculated as unit (inhibition of NBT reduction by 50% per minute) mg^−1^ protein. The activity of peroxisomal enzyme CAT (EC: 1.11.1.6) was determined by monitoring the decline of absorbance observed for 1 min at 240 nm following the procedure of Hossain *et al*.^41^. The reaction recipe contained K-P buffer (50 mM, pH 7.0), H_2_O_2_ (15 mM) and enzyme extract (5 µL) (final volume 700 µL). CAT activity was calculated using an extinction co-efficient of 39.4 M^−1^ cm^−1^. The activity of APX (EC: 1.11.1.11) was assessed according to Nakano and Asada^42^ by recording absorbance change at 290 nm observed for 1 min. The solution mixture was prepared by adding K-P buffer (50 mM, pH 7.0), AsA (0.5 mM), ethylenediaminetetraacetic acid (EDTA, 0.1 mM), H_2_O_2_ (0.1 mM) and enzyme extract (5 µL) (final volume 700 µL). APX activity was estimated with the aid of the extinction co-efficient of 2.8 M^−1^ cm^−1^. Peroxidase (POD, EC: 1.11.1.7) activity was assessed according to the method of Hemeda and Klein^43^. The reaction mixture contained K-P buffer (25 mM, pH 7.0), guaiacol (0.05%), H_2_O_2_ (10 mM) and the enzyme solution (5 µL) (final volume 700 µL). Increase in absorbance was recorded at 470 nm for 1 min, and the extinction co-efficient of 26.6 mM^−1^ cm^−1^ was used for the calculation of POD activity.

The activity of GPX (EC: 1.11.1.9) was estimated according to the protocol of Mahmud *et al*.^44^. The reaction recipe was prepared by adding K-P buffer (100 mM, pH 7.5), EDTA (1 mM), sodium azide (NaN_3_, 1 mM), nicotinamide adenine dinucleotide phosphate (NADPH, 0.12 mM), GSH (2 mM), 1 unit glutathione reductase(GR), H_2_O_2_ (0.6 mM) and enzyme solution (5 µL) (final volume 500 µL). NADPH oxidation was recorded as the change in absorbance observed for 1 min at 340 nm, and the extinction co-efficient of 6.62 mM^−1^ cm^−1^ was used for the calculation of GPX activity. GST (EC: 2.5.1.18) activity was measured following the procedure of Hossain *et al*.^41^. The reaction mixture consisted of Tris-HCl buffer (100 mM, pH 6.5), GSH (1.5 mM), 1-chloro-2,4-dinitrobenzene (CDNB, 1 mM) and enzyme extract (5 µL) (final volume 700 µL). The reaction was started by adding CDNB to the solution, and the absorbance change at 340 nm was recorded for 1 min. The extinction co-efficient of 9.6 mM^−1^ cm^−1^ was used for the calculation of GST activity.

### Contents of proline, total free amino acids, water soluble proteins and soluble sugars

The contents of proline (Pro), total free amino acids and water soluble proteins were appraised according to the protocols of Bates *et al*.^45^, Lee and Takahashi^46^ and Bradford^47^, respectively. The levels of total soluble sugars were colorimetrically assessed following the method of Somogyi^48^.

### Statistical analysis

The obtained data were analyzed by one-way analysis of variance (ANOVA), and the significant variations among the treatments were denoted by different alphabetical letters according to the least significant difference test at *P* < 0.05 using Statistix 10 software. All the data incorporated in the figures and tables are means ± standard errors (SEs) of three biological replicates for individual treatment. Clustering and principal component analysis (PCA) of the data were conducted as previously described by Abdel Latef *et al*.^49^. The biplot was produced with the first two components (PC1 and PC2) that interpreted maximum differences among the datasets.

## Results

### Acetic acid revived phenotypic appearance of mung bean plants under saline conditions

**Figure 1:**
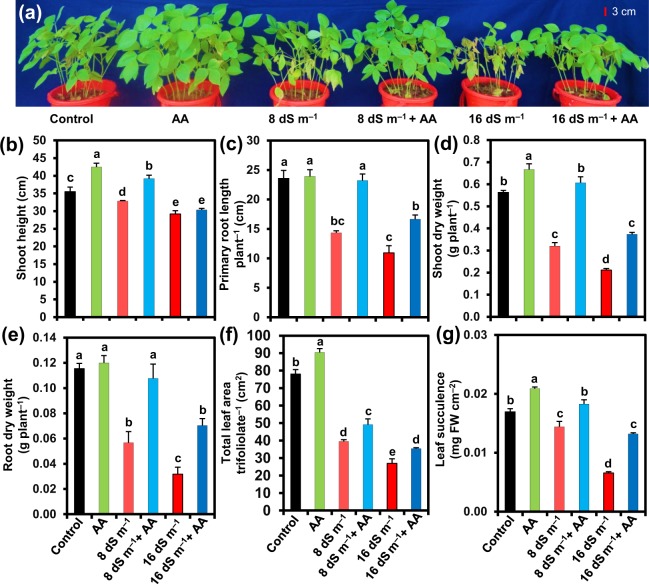
Effects of foliar-applied acetic acid on (**a**) the overall growth, (**b**) shoot height, (**c**) primary root length per plant, (**d**) shoot dry weight per plant, (**e**) root dry weight per plant, (**f**) total leaf area per trifoliolate, and (**g**) leaf succulence of mung bean plants subjected to 0, 8 or 16 dS m^−1^ saline level for 14 days. Values (means ± SEs) of each treatment were attained from three biological replications (*n = *3). Different alphabetical letters above the bars show statistically significant differences (*P* < 0.05) among the treatments, following a least significant difference test. AA, acetic acid; FW, fresh weight.

To analyze the toxic effects of soil salinity on mung bean plants, we applied two levels of seawater at 8 and 16 dS m^−1^ to the pots, and the photographs of mung bean plants were taken at day 14^th^ of salt application. We observed that seawater induced an evident salt stress, which was manifested at phenotypic level in terms of wilting, yellowing of leaves, stunted growth, early senescence, chlorosis, necrosis and even burning of entire leaves (Fig. 1a). On the other hand, spraying of mung bean plants with acetic acid (AA, 20 mM) notably reduced the seawater-induced adverse effects, and improved plant performance when compared with the plants irrigated with seawater only (Fig. 1a). We also noticed that application of external AA to non-stressed mung bean plants showed an incredible improvement in their growth when contrasted with that of control plants (Fig. 1a).

### Acetic acid improved mung bean growth and biomass under salinity

To examine how AA application helped plants overcome salt-induced adverse effects on growth and development, we measured several growth-related parameters, including shoot height, primary root length, DWs of roots and shoots, total leaf area per trifoliolate and leaf succulence under both normal and saline conditions with and without AA foliar application (Fig. 1b–g). In comparison with untreated control, 8 and 16 dS m^−1^ salinity levels markedly decreased shoot height (by 7.44 and 17.78%, respectively), primary root length (by 39.18 and 53.61%, respectively), shoot DW (by 43.15 and 62.51%, respectively), root DW (by 50.87 and 72.43%, respectively), total leaf area per trifoliolate (by 49.42 and 65.39%, respectively) and leaf succulence (by 14.93 and 61.14%, respectively) (Fig. 1b–g). Foliar spray of mung bean plants with AA impressively improved shoot height (by 19.29 and 4.00%), primary root length (by 61.86 and 52.13%), shoot DW (by 89.45 and 76.77%), root DW (by 90.00 and 121.17%), total leaf area per trifoliolate (by 24.52 and 31.09%) and leaf succulence (by 26.62 and 103.38%) at 8 and 16 dS m^−1^ salinity levels, respectively, when compared with the respective plants exposed to seawater alone (Fig. 1b–g). Furthermore, AA-sprayed only plants also exhibited enhanced shoot height, primary root length, shoot DW, root DW, total leaf area per trifoliolate and leaf succulence by 19.81, 1.41, 18.34, 4.05, 15.82 and 23.32%, respectively, over untreated control (Fig. 1b–g).

### Acetic acid regulated the uptake of Na^+^, K^+^ and other minerals under salt stress conditions

**Figure 2:**
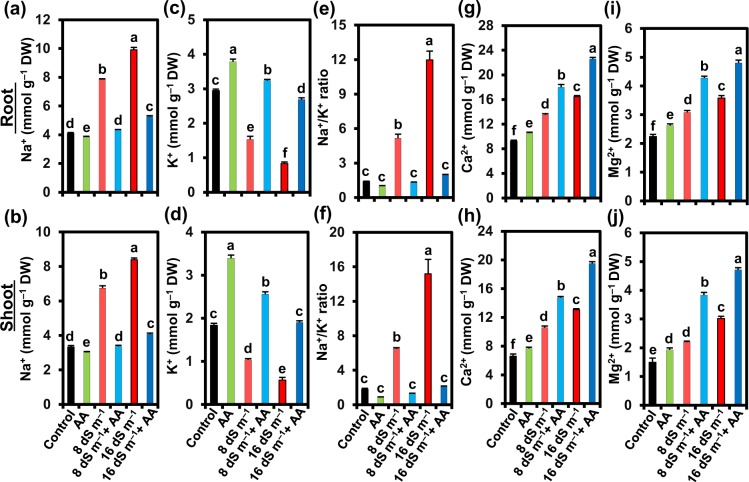
Effects of foliar-applied acetic acid on the levels of (**a**,**b**) Na^+^, (**c**,**d**) K^+^, (**e**,**f**) Na^+^/K^+^ ratio, (**g**,**h**) Ca^2+^, and (**i**,**j**) Mg^2+^ in roots and shoots of mung bean plants subjected to 0, 8 or 16 dS m^−1^ saline level for 14 days. Values (means ± SEs) of each treatment were attained from three biological replications (*n = *3). Different alphabetical letters above the bars show statistically significant differences (*P* < 0.05) among the treatments, following a least significant difference test. AA, acetic acid; DW, dry weight.

The putative role of AA on selective ion uptake capacity of mung bean plants under soil salinity was evaluated by determining the contents of K^+^, Na^+^, Ca^2+^ and Mg^2+^ in both roots and shoots (Fig. 2). Mung bean plants exposed to 8 and 16 dS m^−1^ seawater levels showed a notable increase in the levels of Na^+^ (by 91.38 and 141.87% in roots, and by 102.70 and 152.05% in shoots, respectively), and a significant decline in K^+^ contents (by 48.08 and 71.72% in roots, and by 43.56 and 69.15% in shoots, respectively) in comparison with untreated control (Fig. 2a–d). We also calculated the ratio of Na^+^/K^+^, and found that 8 and 16 dS m^−1^ salinity levels caused remarkable increases in Na^+^/K^+^ ratio in both roots (by 271.25 and 760.10%, respectively) and shoots (by 258.45 and 735.57%, respectively) compared with that in seawater-devoid control plants (Fig. 2e,f). It is worth noting that Ca^2+^ content surprisingly increased by 46.78 and 78.45% in roots, and 60.17 and 98.13% in shoots under 8 and 16 dS m^−1^ saline levels, respectively, in comparison with control plants (Fig. 2g,h). Similarly, treatment with 8 and 16 dS m^−1^ salinity levels led to the increment of Mg^2+^ content by 37.70 and 60.06% in roots, and 47.32 and 102.23% in shoots, respectively, as compared with untreated control plants (Fig. 2i,j).

Notably, application of AA to seawater-treated plants remarkably reduced the Na^+^ content (by 44.90 and 46.96% in roots, and by 50.02 and 51.47% in shoots, respectively), increased the K^+^ content (by 111.55 and 222.40% in roots, and by 146.95 and 236.47% in shoots, respectively), and a resultant decrease of Na^+^/K^+^ ratio (by 74.15 and 83.63% in roots, and by 79.76 and 85.93% in shoots, respectively) (Fig. 2a–f). Additionally, AA foliar spraying to seawater-treated plants further enhanced the Ca^2+^ content (by 33.10 and 37.18% in roots, and by 39.55 and 49.10% in shoots, respectively) and Mg^2+^ content (by 38.96 and 34.17% in roots, and by 74.39 and 56.07% in shoots, respectively) in comparison with that of seawater-treated plants only (Fig. 2g–j). Nevertheless, external supply of exogenous AA to non-stressed plants caused a reduction in Na^+^ content (by 6.42 and 9.31%, respectively) and an enhancement in K^+^ content (by 28.51 and 84.75%, respectively), and a consequent reduction of Na^+^/K^+^ ratio (by 27.19 and 51.02%, respectively), while maintaining the elevated levels of Ca^2+^ (by 14.39 and 16.20%, respectively) and Mg^2+^ (by 17.29 and 29.24%, respectively) in roots and shoots, as compared with that of water-sprayed non-stressed control (Fig. 2a–j).

### Acetic acid modulated gas exchange features in seawater-exposed mung bean plants

**Figure 3:**
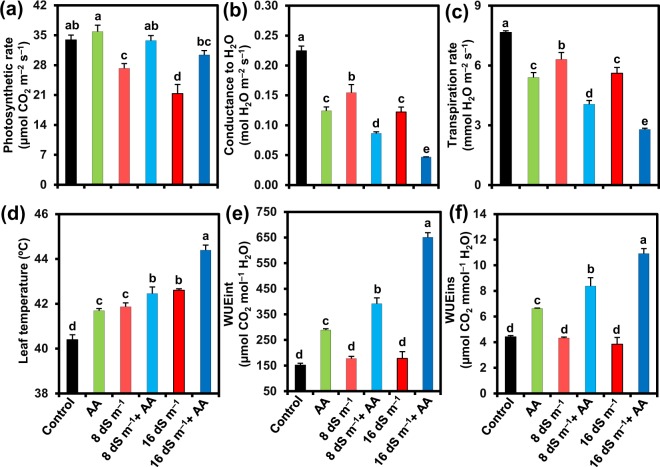
Effects of foliar-applied acetic acid on (**a**) photosynthetic rate, (**b**) stomatal conductance to H_2_O, (**c**) transpiration rate, (**d**) leaf temperature, (**e**) WUEint (intrinsic water use efficiency), and (**f**) WUEins (instantaneous water use efficiency) in leaves of mung bean plants subjected to 0, 8 or 16 dS m^−1^ saline level for 14 days. Values (means ± SEs) of each treatment were attained from three biological replications (*n = *3). Different alphabetical letters above the bars show statistically significant differences (*P* < 0.05) among the treatments, following a least significant difference test. AA, acetic acid.

Photosynthetic features of mung bean plants exposed to salt stress were appraised by determining photosynthetic rate (Pn), stomatal conductance to H_2_O (g_s_), transpiration rate (E), leaf temperature (LT), intrinsic water use efficiency (WUEint) and instantaneous water use efficiency (WUEins) of the leaves. Exposure of mung bean plants to 8 and 16 dS m^−1^ saline levels caused a reduction of Pn by 19.59 and 37.07%, g_s_ by 31.09 and 45.56%, E by 17.66 and 26.78%, and WUEins by 2.13 and 13.00%, but an enhancement of LT by 3.64 and 5.47%, and WUEint by 17.30 and 17.35%, respectively, relative to control plants (Fig. 3). On the other hand, application of AA to the plants stressed with 8 and 16 dS m^−1^ noticeably increased Pn by 23.78 and 42.64%, WUEint by 120.53 and 266.37%, WUEins by 93.66 and 183.60%, while slightly elevating LT by 1.41 and 4.19%, respectively, when compared with those of the plants stressed with salt only. AA spraying resulted in further decrement of g_s_ by 44.17 and 61.77%, and E by 35.76 and 50.25% in 8 and 16 dS m^−1^ salt-stressed plants, respectively, relative to that observed in salt-stressed only plants (Fig. 3). Under salt-free conditions, AA-supplied plants displayed an enhancement of Pn, LT, WUEint and WUEins by 5.72, 3.21, 90.75 and 50.11%, respectively, while a reduction in g_s_ and E by 44.67 and 29.52%, respectively, in comparison with control (Fig. 3).

### Acetic acid protected photosynthetic pigments in mung bean leaves under soil salinity

**Figure 4:**
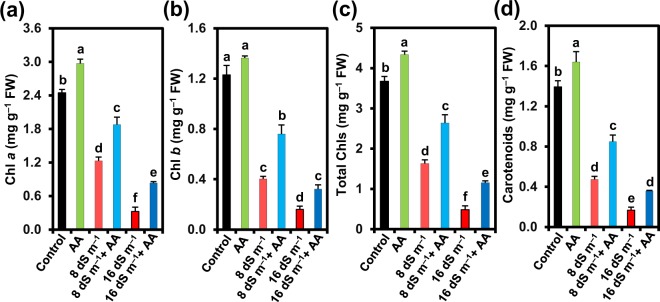
Effects of foliar-applied acetic acid on the contents of (**a**) chlorophyll (Chl) *a*, (**b**) Chl *b*, (**c**) total Chls, and (**d**) carotenoids in leaves of mung bean plants subjected to 0, 8 or 16 dS m^−1^ saline level for 14 days. Values (means ± SEs) of each treatment were attained from three biological replications (*n = *3). Different alphabetical letters above the bars show statistically significant differences (*P* < 0.05) among the treatments, following a least significant difference test. AA, acetic acid; FW, fresh weight.

To assess the protective roles of exogenous AA on the photosynthetic performance under salt stress, the levels of photosynthetic pigments like Chl *a*, Chl *b*, total Chls and Cars in salt-exposed mung bean leaves were determined (Fig. 4). In comparison with the untreated control, there was a decrease in Chl *a* content by 49.68 and 86.73%, Chl *b* content by 67.20 and 86.92%, total Chls content by 55.54 and 86.79%, and Cars content by 65.98 and 87.94% in the plants exposed to 8 and 16 dS m^−1^ salinity stresses, respectively (Fig. 4). In contrast, spraying of AA protected photosynthetic pigments from salinity-induced toxic effects, as evident by the observed improved contents of Chl *a* (52.62 and 156.29%), Chl *b* (88.24 and 100.28%), total Chls (61.41 and 137.73%) and Cars (79.27 and 112.68%) in responses to 8 and 16 dS m^−1^ seawater doses, respectively, when compared with salt-treated only plants (Fig. 4). Nonetheless, non-stressed mung bean plants sprayed with AA also showed increased contents of Chl *a* (by 21.51%), Chl *b* (by 11.01%), total Chls (by 18.00%) and Cars (by 17.77%), when compared with water-sprayed non-stressed control (Fig. 4).

### Acetic acid reduced seawater-induced oxidative damage in mung bean plants

**Figure 5:**
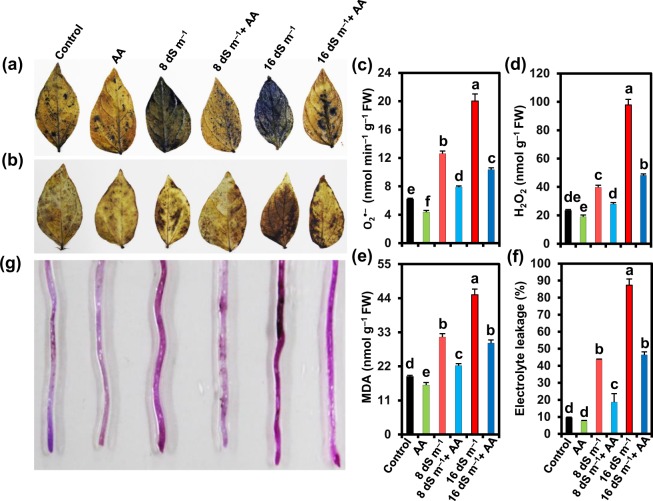
Effects of foliar-applied acetic acid on reactive oxygen species accumulations in leaves and membrane damage in roots of mung bean plants subjected to 0, 8 or 16 dS m^−1^ saline level for 14 days. (**a**) Superoxide (O_2_^•−^) stained with nitroblue tetrazolium (NBT), (**b**) hydrogen peroxide (H_2_O_2_) stained with diaminobenzidine (DAB). Quantification of (**c**) O_2_^•−^, (**d**) H_2_O_2_ and (**e**) malondialdehyde (MDA) in leaves, and (**f**) electrolyte leakage levels in shoots of mung bean plants. (**g**) Schiff’s test for lipid peroxidation in roots. Values (means ± SEs) of each treatment were attained from three biological replications (*n = *3). Different alphabetical letters above the bars show statistically significant differences (*P* < 0.05) among the treatments, following a least significant difference test. AA, acetic acid; FW, fresh weight.

Salt exposure elicited an enhanced accumulation of ROS like O_2_^•−^ and H_2_O_2_ as observed by more scattered dark blue spots for O_2_^•−^ (Fig. 5a), and more dark brown polymerization patches for H_2_O_2_ (Fig. 5b) in leaves of seawater-treated mung bean plants than in untreated control. Conversely, AA-sprayed salt-stressed plants displayed lower accumulations of O_2_^•−^ and H_2_O_2_ in the leaves relative to that in salt-stressed alone plants (Fig. 5a,b). More specifically, we noted that the leaves of mung bean plants subjected to 8 and 16 dS m^−1^ salinity levels significantly enhanced the contents of O_2_^•−^ by 104.01 and 224.09%, and H_2_O_2_ by 70.81 and 320.30%, respectively, compared with non-stressed control (Fig. 5c,d). However, AA application reduced the contents of O_2_^•−^ by 37.39 and 48.31%, and H_2_O_2_ by 29.78 and 50.79% in salt-exposed plants (8 and 16 dS m^−1^ seawater levels, respectively) in comparison with the plants stressed with salt only (Fig. 5c,d). In addition, mung bean plants subjected to 8 and 16 dS m^−1^ salinity levels exhibited an increased level of MDA by 68.57 and 141.78%, and EL by 361.46 and 823.83%, respectively, in correspondence with non-stressed control (Fig. 5e,f). Addition of AA to salt-stressed plants played a decisive role in reducing the levels of MDA (by 29.26 and 34.58%, respectively) and EL (by 57.26 and 46.93%, respectively) compared with the plants exposed to 8 or 16 dS m^−1^ seawater level only (Fig. 5e,f). AA application to plants grown under normal conditions also reduced the levels of O_2_^•−^, H_2_O_2_, MDA and EL by 29.20, 18.32, 14.60 and 19.67%, respectively, in the leaves as compared with that of untreated control (Fig. 5c–f). To examine whether roots also suffered ROS-mediated oxidative damage, we investigated the membrane integrity of roots by a Schiff staining, and found increased purple color intensity in roots of mung bean plants grown on saline soil (Fig. 5g). On the other hand, roots of AA-sprayed salt-treated plants showed a decreased uptake of Schiff’s reagent, as visualized by the remarkably lower intensity of purple color, when compared with salt-treated only plants (Fig. 5g).

**Figure 6:**
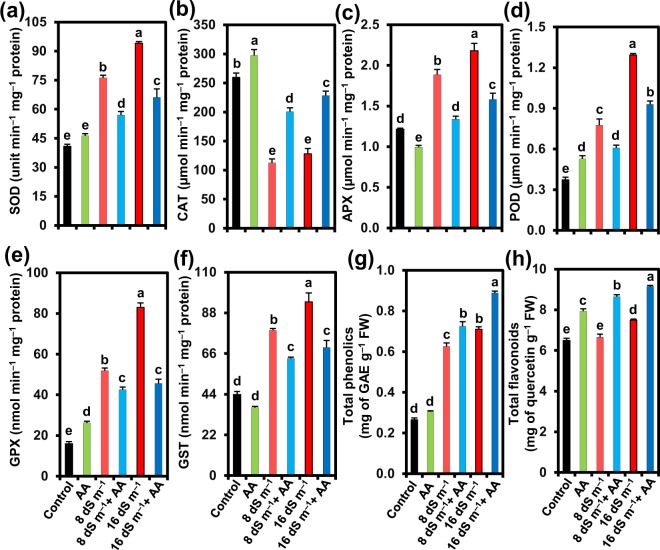
Effects of foliar-applied acetic acid on the activities of (**a**) SOD (superoxide dismutase), (**b**) CAT (catalase), (**c**) APX (ascorbate peroxidase), (**d**) POD (peroxidase), (**e**) GPX (glutathione peroxidase), (**f**) GST (glutathione *S*-transferase), and (**g**) total phenolic and (**h**) total flavonoid contents in leaves of mung bean plants subjected to 0, 8 or 16 dS m^−1^ saline level for 14 days. Values (means ± SEs) of each treatment were attained from three biological replications (*n = *3). Different alphabetical letters above the bars show statistically significant differences (*P* < 0.05) among the treatments, following a least significant difference test. AA, acetic acid; FW, fresh weight; GAE, gallic acid equivalent.

Next, we determined the activities of SOD, CAT, POD, APX, GPX and GST, and the levels of total phenolics and flavonoids in the leaves of mung bean to appraise their roles in ROS detoxifications in the presence or absence of AA under both normal and saline conditions (Fig. 6). Salt stress at 8 and 16 dS m^−1^ increased the activities of SOD by 86.80 and 130.46%, APX by 55.14 and 79.51%, POD by 108.22 and 247.02%, GPX by 224.35 and 418.84%, and GST by 79.43 and 114.39%, while reducing CAT activity by 56.65 and 50.67%, respectively, as compared with corresponding values in control plants (Fig. 6a–f). Foliar application of AA caused significant enhancement of CAT activity by 78.24 and 78.21% at 8 and 16 dS m^−1^ salinity levels, respectively, as compared with that of plants faced with seawater-induced stress only (Fig. 6b). However, the activities of other antioxidant enzymes, including SOD, APX, POD, GPX and GST, did not increase further after the supplementation of AA to the salt-stressed plants (Fig. 6a,c–f). Nonetheless, under normal conditions, AA application increased the activities of SOD, CAT, POD and GPX by 13.51, 14.50, 41.50 and 63.60%, respectively, whereas APX and GST activities reduced by 18.24 and 16.14%, respectively, as compared with that of untreated control plants (Fig. 6a–f).

Plants treated with 8 and 16 dS m^−1^ salinity levels showed an enhancement in the contents of total phenolics by 136.31 and 168.01%, and total flavonoids by 2.46 and 15.45%, respectively, over that of untreated control plants (Fig. 6g,h). Application of AA further augmented the levels of total phenolics by 15.72 and 25.11%, and total flavonoids by 29.96 and 22.01% in the leaves of mung bean stressed with seawater of 8 and 16 dS m^−1^, respectively, when compared with that of salt-stressed only plants (Fig. 6g,h). In the absence of seawater treatment, AA supplementation increased total phenolics and total flavonoids by 15.35 and 22.23%, respectively, relative to that of control plants (Fig. 6g,h).

### Acetic acid elevated shoot water status and osmoprotectant accumulations in salinity- exposed mung bean plants

Shoot RWC of mung bean plants markedly decreased by 21.98 and 31.15% at 8 and 16 dS m^−1^ of salinity levels, respectively, as compared with that of untreated control. In contrast, spraying salt-stressed plants with AA greatly rescued RWC, almost to the level of water-irrigated control, by increasing shoot RWC by 28.70 and 30.57% at 8 and 16 dS m^−1^ salinity levels, respectively, compared with AA unsprayed salt-stressed plants (Table 1). To relate the water status with the osmoprotectant levels, we estimated the contents of Pro, total free amino acids, water soluble proteins and total soluble sugars (Table 1). Imposition of 8 and 16 dS m^−1^ salt stress led to an enhancement in the contents of Pro by 164.15 and 241.51%, total free amino acids by 35.10 and 130.14%, water soluble proteins by 7.41 and 13.73%, and total soluble sugars by 50.77 and 84.72%, respectively, as compared with that of untreated control plants (Table 1). On the other hand, supplementation of AA to the plants subjected to 8 and 16 dS m^−1^ resulted in an increased accumulation of Pro by 117.14 and 144.75%, total free amino acids by 51.79 and 68.74%, water soluble proteins by 10.06 and 15.33%, and total soluble sugars by 42.91 and 69.64%, respectively, in comparison with the plants exposed to the same levels of salt stress without AA spraying (Table 1). AA application to unstressed plants increased the contents of Pro, total free amino acids, water soluble proteins and total soluble sugars by 33.96, 17.21, 19.44 and 26.07%, respectively, compared with that of untreated control plants (Table 1).

**Table 1:** Effects of foliar-applied acetic acid on the levels of shoot relative water content, proline, total free amino acids, water soluble proteins, and total soluble sugars in mung bean plants subjected to 0, 8 or 16 dS m^−1^ saline level for 14 days.

Treatments	Shoot relative water content (%)	Proline (µmol g^−1^ FW)	Total free amino acids (mg g^−1^ FW)	Water soluble proteins (mg g^−1^ FW)	Total soluble sugars (mg g^−1^ FW)
Control	79.58 ± 1.28^a^	0.53 ± 0.02^d^	8.66 ± 0.08^d^	6.48 ± 0.20^b^	124.51 ± 1.42^e^
AA	81.55 ± 0.81^a^	0.71 ± 0.02^d^	10.15 ± 0.11^cd^	7.74 ± 0.54^ab^	156.97 ± 5.59^de^
8 dS m^−1^	62.09 ± 1.30^c^	1.40 ± 0.03^c^	11.70 ± 0.23^c^	6.96 ± 0.18^b^	187.72 ± 10.58^d^
AA + 8 dS m^−1^	79.91 ± 0.86^a^	3.04 ± 0.30^b^	17.76 ± 0.54^b^	7.66 ± 0.20^ab^	268.27 ± 9.05^b^
16 dS m^−1^	54.79 ± 2.66^d^	1.81 ± 0.06^c^	19.93 ± 0.50^b^	7.37 ± 0.54^ab^	230.00 ± 2.60^c^
AA + 16 dS m^−1^	71.54 ± 0.94^b^	4.43 ± 0.34^a^	33.63 ± 1.55^a^	8.50 ± 0.88^a^	390.18 ± 21.06^a^

Values (means ± SEs) of each treatment were attained from three biological replications (*n* = 3). Values within column with different alphabetical letters show statistically significant differences (*P* < 0.05) among the treatments, following a least significant difference test. AA, acetic acid; FW, fresh weight.

### Visualization of data with clustered heatmap and unveiling the treatment-variable relationship by PCA

**Figure 7:**
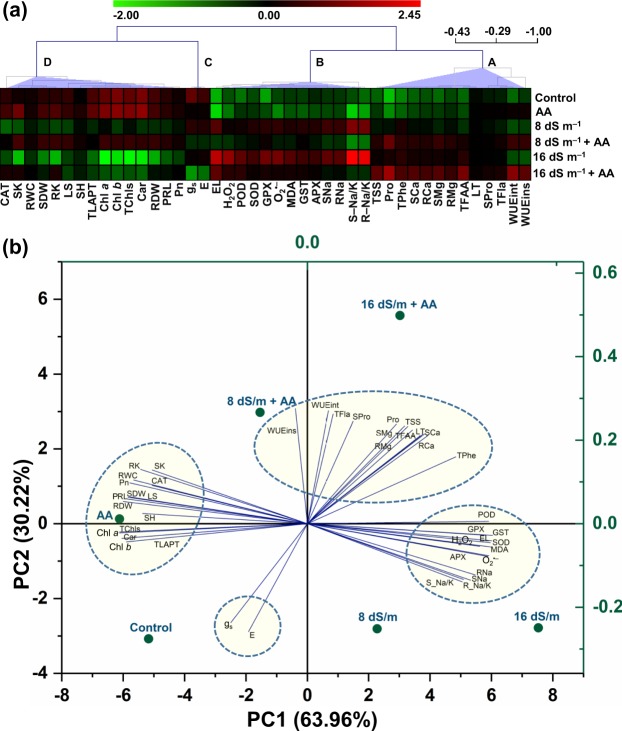
(**a**) To visualize all of the collected data at a glance with color intensity, clustered heatmap was prepared by normalization of mean values of different parameters using the MeV software (version 4.9.0). Four distinct clusters (A–D) were identified at variable levels. Color scale indicates the changing trend of the normalized mean values of different parameters under different treatments. (**b**) Principal component analysis (PCA) was conducted to unveil the relationships between treatments and variable levels. The lines derived from the middle point of the biplot exhibited positive or negative interactions of multiple physio-biochemical parameters with different treatments. The small or bigger angle connecting the variable and treatment to the origin indicates strong or weak association, respectively, between treatment and variables. The variables included SH (shoot height), PRL (primary root length per plant), SDW (shoot dry weight), RDW (root dry weight), TLAPT (total leaf area per trifoliolate), LS (leaf succulence), SNa (shoot Na^+^ content), RNa (root Na^+^ content), SK (shoot K^+^ content), RK (root K^+^ content), S-Na/K (shoot Na^+^/K^+^ ratio), R-Na/K (root Na^+^/K^+^ ratio), SCa (shoot Ca^2+^ content), RCa (root Ca^2+^ content), SMg (shoot Mg^2+^ content), RMg (root Mg^2+^ content), Pn (photosynthetic rate), g_s_ (conductance to H_2_O), E (transpiration rate), LT (leaf temperature), WUEint (intrinsic water use efficiency), WUEins (instantaneous water use efficiency), Chl *a* (chlorophyll *a*), Chl *b* (chlorophyll *b*), TChls (total chlorophylls), Car (carotenoids), O_2_^•−^ (superoxide), H_2_O_2_ (hydrogen peroxide), MDA (malondialdehyde), EL (electrolyte leakage), SOD (superoxide dismutase), CAT (catalase), APX (ascorbate peroxidase), POD (peroxidase), GPX (glutathione peroxidase), GST (glutathione *S*-transferase), TPhe (total phenolics), TFla (total flavonoids), RWC (relative water content), Pro (proline), TFAA (total free amino acids), SPro (water soluble proteins), and TSS (total soluble sugars). AA, acetic acid.

In order to visualize the collected data at a glance with color intensity, we generated a clustered heatmap (Fig. 7a). Variables of cluster-A and -B depicted an increasing trend upon exposure of plants to 8 or 16 dS m^−1^ of salt stress, in comparison with seawater-devoid control treatment, aside from the diminishing patterns of WUEins under both doses of seawater (Fig. 7a). In contrast, cluster-C and -D variables obtained at 8 or 16 dS m^−1^ of salt stress exhibited diminishing patterns when compared with untreated control plants (Fig. 7a). Supplementation of AA as a foliar spray to plants exposed to either gradient of salinity resulted in an increasing trend in the variables of cluster-A and -D, in contrast to that of plants confronted 8 or 16 dS m^−1^ of salt stress only (Fig. 7a). On the other hand, plants supplemented with AA under 8 and 16 dS m^−1^ salt stresses demonstrated a diminishing trend in the variables of cluster-B and -C, when equated with the plants exposed to corresponding level of salt stress alone (Fig. 7a). Plants sprayed with AA under non-stressed condition displayed an increasing trend in all variables of cluster-A and -D, relative to that of water-sprayed unstressed control plants (Fig. 7a). Conversely, variables of cluster-B and -C showed a decreasing trend in AA-sprayed non-stressed plants, except the increasing patterns of SOD, CAT, POD and GPX activities being observed in the cluster-B, in comparison with that of water-sprayed unstressed control plants (Fig. 7a). Afterwards, PCA was performed to find the correlations between treatments and different variables (Fig. 7b). The PC1 and PC2 values collectively interpreted 94.18% of data variability. Rather than each and every other treatment, PCA delineated that variables of cluster-A were moderately and strongly connected with AA-supplemented 8 and 16 dS m^−1^ saline levels, respectively, aside from the strong association of WUEins being observed with AA-applied 8 dS m^−1^ saline level (Fig. 7a,b). In contrast, cluster-B variables were more strongly associated with salt treatments of 8 and 16 dS m^−1^, when compared with that observed between cluster-B variables and every other single treatment (Fig. 7a,b). Features of cluster-C and -D were intently interlinked with untreated control and AA-supplemented only plants, respectively, as opposed to every other treatment (Fig. 7a,b).

## Discussion

Growth inhibition and biomass reduction are often considered as the obvious outcomes of salinity-induced negative effects in plants^50^. In the current study, soil salinity-mediated deleterious effects were manifested as the reductions of shoot height, primary root length and total leaf area per trifoliolate, which together contributed to the biomass loss of mung bean (Fig. 1a–f), as they were also reported in other legumes like faba bean (*Vicia faba*)^51^ and chickpea (*Cicer arietinum*)^52^. On the other hand, foliar-applied AA recuperated the growth performance of salt-stressed mung bean plants as evident by their improved phenotypic appearance and growth-associated features, including shoot height, primary root length, total leaf area per trifoliolate and biomass (Fig. 1a–f). These findings were further supported by the results of PCA, which indicated that salt-stressed mung bean plants that received AA displayed a lower extent of negative relationship with growth-related features in comparison with salt-exposed only plants (Fig. 7b), implying the contribution of AA to the alleviation of salinity-induced negative effects on growth-related attributes.

In-depth investigations on mung bean response to salt stress revealed that the salt-induced poor growth performance of mung bean plants (Fig. 1) might be associated with ionic stress due to a notable accumulation of toxic Na^+^, and a significant reduction of the levels of beneficial K^+^ in both shoots and roots (Fig. 2a–d). Our results also manifested in PCA by showing strong and positive correlations between salinity and Na^+^ uptake levels (Fig. 7b). AA supplementation to salt-stressed plants increased K^+^, Ca^2+^ and Mg^2+^ contents, and reduced Na^+^ content as well as Na^+^/K^+^ ratio in both shoots and roots (Fig. 2), which provided an indirect but strong indication that AA helped in effective sequestration of Na^+^ into the vacuoles as further endorsed by the enhanced leaf succulence (Fig. 1g). Succulence facilitates cellular and intracellular sequestrations of toxic Na^+^ ions, thereby restricting excessive Na^+^ accumulation in metabolically active compartments of the cells^53–55^. Our results also showed that AA assisted in the maintenance of K^+^ homeostasis, which has a crucial role in cell expansion and effectual Na^+^ compartmentalization in vacuoles, as well as in stabilization of protein structures and optimization of metabolic functions under ambient salt stress^54,56,57^. Furthermore, augmented divalent cations (Ca^2+^ and Mg^2+^) might also contribute to Chl synthesis, protein synthesis, enzyme activation, protection of membrane structure, signal transduction, blocking the non-selective cation channels and outward-rectifying K^+^ efflux channels, while activating the salt overly sensitive pathways, and enabling efficient K^+^ movement toward photosynthetically active shoot tissues to maintain low Na^+^/K^+^ ratio for optimal photosynthetic rate^58–60^. Our results, therefore, suggested that exogenous AA could ameliorate the salt-induced ion toxicity in mung bean plants by effective Na^+^ sequestration. Furthermore, AA application also promoted the uptake of Ca^2+^, Mg^2+^ and K^+^, resulting in improved cell membrane integrity, nutritional balance and visual appearance of salt-stressed mung bean plants (Figs 1a, and 2c,d,g–j). Even control plants spraying with AA also exhibited lower accumulation of Na^+^, and enhanced uptake of K^+^, Ca^2+^ and Mg^2+^, which suggested that AA might have contributed to the selective nutrient uptake capacity of mung bean roots that helped boosting shoot growth and biomass production (Figs 1b,d and 2).

Results of gas exchange parameters (Fig. 3) revealed that salt stress perturbed photosynthetic performance, which was also coincided with the findings related to growth inhibition and biomass reduction (Fig. 1). In contrast, application of AA to salt-stressed mung bean plants improved the photosynthetic rate, despite having declined stomatal conductance, reduced transpiration rate and elevated leaf temperature (Fig. 3a–d). Treatment of mung bean salt-stressed plants with AA prevented the decline of the photosynthetic pigment contents, including Chl *a*, Chl *b*, total Chl and Car contents (Fig. 4), which might contribute to their enhanced photosynthetic rate as these pigments are directly involved in capturing light energy and invariably associated with photosynthetic performance^61^. Furthermore, the improved photosynthetic rate in AA-supplemented mung bean plants under salt stress might be resulted from the ability of AA to enhance RWC (Table 1) and increase leaf area (Fig. 1f), which could improve the capture of sunlight for better photosynthesis^13,62^. The increased photosynthetic rate in AA-supplied salt-treated plants clearly indicated that AA helped plants to use limited assimilates for maximum efficiency of photosynthesis. Indeed, salt-stressed mung bean plants supplemented with AA not only maintained higher RWC but also more effectively utilized the limitedly available water to produce more biomass than salt-stressed only plants as indicated by their higher WUEint and WUEins (Table 1; Fig. 3e,f). Accordingly, our PCA results endorsed that seawater-treated mung bean plants supplemented with AA had a stronger positive correlation with WUEint and WUEins than seawater-stressed only mung bean plants (Fig. 7b). In support of our findings, a recent study has reported that AA treatment increased drought tolerance of cassava (*Manihot esculenta*) by maintaining higher RWC, increased leaf temperature, and decreased stomatal conductance and transpiration rate through the ABA-regulated stomatal closing^63^. Based on these results, it is plausible to suggest that under stress, application of exogenous AA can maintain photosynthetic efficiency by enhancing photosynthetic pigment contents, RWC and leaf area, as well as improve WUE by reducing stomatal opening and transpiration to decrease water loss and the flow of toxic ions into the transpiration stream.

In the present study, mung bean plants subjected to seawater-induced salinity exhibited significant accumulations of O_2_^•−^ and H_2_O_2_, and an associated increase in MDA level in the leaves (Fig. 5a–e). Salt stress also caused membrane damage as indicated by the increased levels of EL in the leaves and greater lipid peroxidation in the roots of mung bean plants (Fig. 5f,g). Similar to our results, compelling reports have suggested that salt stress imposed a great burden on plant metabolism due to excessive accumulation of Na^+^, resulting in oxidative stress to cellular constituents^30,64^. More importantly, we obtained evidence that foliar-applied AA inhibited ROS accumulations and protected membrane integrity in salt-treated mung bean plants (Fig. 5a–f). These results indicated that AA might ease mung bean suffering from salt stress by diminishing oxidative damage. Accordingly, our PCA results also demonstrated that foliar spraying of AA to the salt-stressed mung bean plants had a positive correlation with O_2_^•−^, H_2_O_2_, MDA and electrolyte leakage, but the correlation extended to a smaller level in comparison with seawater-stressed only mung bean plants (Fig. 7b).

A number of earlier research reports claimed that stimulation of antioxidant capacity following salt treatment is considered as the integral part of plant defense mechanisms to combat ROS toxicity under saline conditions^30,40,65^. We observed that salt treatments alone led to a significant enhancement of the activities of SOD, APX, POD, GPX and GST, but not CAT activity, in a dose-dependent manner (Fig. 6a–f). AA supplementation enhanced the activity of CAT only (Fig. 6b), suggesting that AA activated CAT to defend mung bean plants against salinity-induced oxidative stress. This observation is in line with previous study, where the application of exogenous AA increased the expression levels of *catalase 1* and *catalase 2* genes that play a vital role in maintaining appropriate H_2_O_2_ levels for better survival of cassava plants to drought^63^. Taken together, reduced accumulation of Na^+^ led to decreased oxidative stress effects on AA-supplied salt-stressed plants, as indicated by much lower accumulations of ROS (Fig. 5a–d), which ultimately determined the induction levels of antioxidant enzyme activities. The safeguarding role of AA in CAT activity implied that AA alleviated oxidative stress by protecting the activity of CAT (Fig. 6b), which is largely recognized as a potential detoxifier of H_2_O_2_, especially when H_2_O_2_ accumulates in large quantities under stress conditions^66^. Additionally, it was also evidenced in this study that AA contributed to the removal of ROS by enhancing the levels of non-enzymatic antioxidants like total phenolics and flavonoids (Fig. 6g,h), which are well-known quenchers of ROS^67–71^.

Like oxidative stress, osmotic stress is another adverse component of salt toxicity in susceptible plants^72^. To overcome osmotic stress, plants synthesize and accumulate a range of compatible solutes for osmotic adjustments^73,74^. Our results revealed that salt stress led to significant accumulations of Pro, total free amino acids, water soluble proteins and total soluble sugars in mung bean plants, and spraying AA to salt-stressed plants further boosted their contents (Table 1). In line with this finding, results of PCA indicated stronger positive associations between AA-sprayed salt-stressed plants and the content of these osmoprotectants than that observed between salt-stressed only plants and the same parameters (Fig. 7b). The enhanced accumulation of Pro in AA-supplied salt-stressed plants might contribute to shielding of photosynthetic machinery, quenching of ROS, and stabilizing membranes, proteins and enzymes^6,75,76^, whereas an increase in total free amino acids enables the plants to meet the increasing demand of amino acids during protein metabolisms^13,54^. The accumulation of soluble proteins in mung bean plants under salt stress might play the pivotal role in osmotic adjustment and can provide a source of nitrogen that is re-utilized after the stress is diminished^76,77^. Positive correlations between increased salt tolerance and accumulations of Pro, soluble proteins and total free amino acids have also been reported by Ahmad *et al*.^76^ in *C. arietinum* and Abdel Latef *et al*.^6^ in *V. faba*. Furthermore, increasing total soluble sugar contents by AA might result from AA-mediated enhanced photosynthetic rate, which subsequently contributed to a better growth performance of mung bean plants under salt stress conditions. Soluble sugars act as important osmolytes playing multiple functions in plants, including restriction of water loss and Chl destruction, regulation of cell division and expansion, scavenging of ROS, stabilization of proteins and membrane structure, Pro accumulation, maintenance of osmotic and ion homeostasis, and transcriptional regulation of certain genes^78,79^.

In summary, the current study provides strong lines of evidence that AA successfully ameliorated salt stress-induced growth inhibition and biomass loss by regulating several physiological and biochemical mechanisms, including (i) acceleration of leaf succulence, (ii) attenuation of uptake and accumulation of toxic Na^+^, (iii) retention of ion homeostasis and photosynthetic capacity, (iv) protection of photosynthetic pigments, (v) reduction of oxidative stress through the inhibition of ROS and MDA accumulations, (vi) maintenance of membrane integrity, (vii) enhancement of CAT activity, and the contents of phenolics and flavonoids, and (viii) stimulation of osmoprotectant productions. Taken together, these results suggest that AA supplementation could be a viable cost-effective technology that can be employed for the mitigation of salt stress-caused adverse effects on crop performance to ensure sustainable agriculture in saline-prone areas. However, further studies at the field level using different modes of AA applications to a range of crop species under various regimes of salinity should be needed to ascertain the beneficial roles of AA in the management of salinity. Additionally, it would be fascinating to identify whether AA application positively alters seed biochemical and nutrient contents of mung bean that may help us deal with undernutrition problems of the people living in many developing countries.
